# Segmentation-based quantitative measurements in renal CT imaging using deep learning

**DOI:** 10.1186/s41747-024-00507-4

**Published:** 2024-10-09

**Authors:** Konstantinos Koukoutegos, Richard ’s Heeren, Liesbeth De Wever, Frederik De Keyzer, Frederik Maes, Hilde Bosmans

**Affiliations:** 1 KU Leuven, Department of Imaging and Pathology, Division of Medical Physics, Herestraat 49, 3000 Leuven, Belgium; 2 UZ Leuven, Department of Radiology, Herestraat 49, 3000 Leuven, Belgium; 3 KU Leuven, Department of Electrical Engineering, ESAT/PSI, 3000 Leuven, Belgium

**Keywords:** Abdomen, Artificial intelligence, Deep learning, Kidney, Tomography (x-ray computed)

## Abstract

**Background:**

Renal quantitative measurements are important descriptors for assessing kidney function. We developed a deep learning-based method for automated kidney measurements from computed tomography (CT) images.

**Methods:**

The study datasets comprised potential kidney donors (*n* = 88), both contrast-enhanced (Dataset 1 CE) and noncontrast (Dataset 1 NC) CT scans, and test sets of contrast-enhanced cases (Test set 2, *n* = 18), cases from a photon-counting (PC)CT scanner reconstructed at 60 and 190 keV (Test set 3 PCCT, *n* = 15), and low-dose cases (Test set 4, *n* = 8), which were retrospectively analyzed to train, validate, and test two networks for kidney segmentation and subsequent measurements. Segmentation performance was evaluated using the Dice similarity coefficient (DSC). The quantitative measurements’ effectiveness was compared to manual annotations using the intraclass correlation coefficient (ICC).

**Results:**

The contrast-enhanced and noncontrast models demonstrated excellent reliability in renal segmentation with DSC of 0.95 (Test set 1 CE), 0.94 (Test set 2), 0.92 (Test set 3 PCCT) and 0.94 (Test set 1 NC), 0.92 (Test set 3 PCCT), and 0.93 (Test set 4). Volume estimation was accurate with mean volume errors of 4%, 3%, 6% mL (contrast test sets) and 4%, 5%, 7% mL (noncontrast test sets). Renal axes measurements (length, width, and thickness) had ICC values greater than 0.90 (*p* < 0.001) for all test sets, supported by narrow 95% confidence intervals.

**Conclusion:**

Two deep learning networks were shown to derive quantitative measurements from contrast-enhanced and noncontrast renal CT imaging at the human performance level.

**Relevance statement:**

Deep learning-based networks can automatically obtain renal clinical descriptors from both noncontrast and contrast-enhanced CT images. When healthy subjects comprise the training cohort, careful consideration is required during model adaptation, especially in scenarios involving unhealthy kidneys. This creates an opportunity for improved clinical decision-making without labor-intensive manual effort.

**Key Points:**

- Trained 3D UNet models quantify renal measurements from contrast and noncontrast CT.
- The models performed interchangeably to the manual annotator and to each other.
- The models can provide expert-level, quantitative, accurate, and rapid renal measurements.

**Graphical Abstract:**

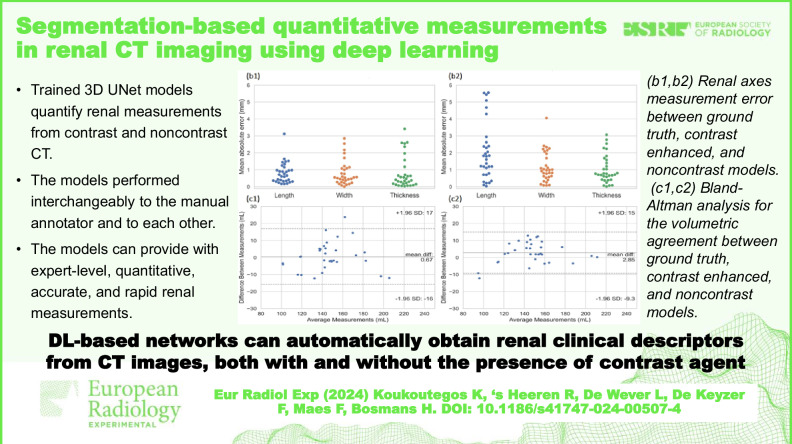

## Background

Renal quantitative measurements such as kidney length and volume are important clinical indicators used daily in radiology to describe morphological characteristics, assess renal functionality, determine the presence and/or progression of renal disease, and evaluate an individual’s eligibility as a kidney donor [1–4]. In kidney donor-recipient matching pairs, kidney size is of great importance [5]. Graft volume has been shown to correlate significantly with improved transplantation outcomes both in terms of glomerular filtration rate and 1-year serum creatinine level [6–9].

Ultrasound imaging is noninvasive, can be acquired fast, and does not expose the subject to ionizing radiation, making it the preferred imaging modality for obtaining such measurements [1, 10–12]. Nevertheless, the suitability of ultrasound has been controversial because of its two-dimensional nature and the fact that it requires basic geometric assumptions about renal morphology [13]. Previous studies have demonstrated that renal measurements in computed tomography (CT) provide more consistent results compared to ultrasound [14]. The inclusion of three-dimensional (3D) information from CT scanners stabilized the measurements even further [15].

Manual CT measurements, although accurate, are labor-intensive and subject to interobserver and intraobserver variability [16, 17]. Therefore, in many studies, renal volume estimates have been obtained from simple ellipsoid fitting to the kidney [6, 7, 10, 18, 19]. This approach provides a significant speed-up in the measurement process but leads to suboptimal results as the kidneys are not ellipsoid [1]. Furthermore, this method suffers from observer variability because the three axes of each kidney must be defined manually [20].

Deep learning-based renal volume measurements have also been studied, especially as part of the Kidney and Kidney Tumor Segmentation Challenge (KiTS) (https://kits-challenge.org/kits23/). Multiple teams have either participated in the challenge or used its dataset to obtain accurate measurements of the kidney and the kidney tumor [21–26]. Although the performance reached that of the manual annotators, the challenge dataset included the renal sinus fat that does not contain functional renal tissue [1]. Such a dataset is not entirely representative of real-world settings, where measuring the actual renal parenchyma is often considered during transplantation planning. Additionally, KiTS focuses only on contrast-enhanced (CE) CT that does not accurately resemble real clinical settings. Especially in patients with kidney impairment, the toxicity risks of using intravenous iodinated contrast agents are taken into consideration, and patients are scanned, when possible, without contrast administration [27]. In addition, it is known that contrast agents slightly increase the size of the kidneys [14].

Therefore, we aimed to develop a deep learning-based method to obtain quantitative renal measurements for both CE and noncontrast (NC) CT images.

## Methods

### Datasets

This retrospective study used different datasets, with due ethical clearance by the ethical committee at the University Hospital of Leuven (internal reference number S66718). The first one (Dataset 1) comprised CT images of kidney donors who, from February 2018 to April 2022, underwent at our institution a standard CT examination with NC scans followed by CE phases, in order to assess eligibility to donate (acquisition protocol described in Supplementary material “Dataset 1 protocol”). Of note, part of Dataset 1 has been use as a first test set (Test set 1). The second dataset (Test set 2) is composed of CE images and was randomly sampled from our institution’s Picture archiving and communications system. The selection process included images scanned using identical protocol to the one of Dataset 1, but without necessarily images of healthy kidneys, in an attempt to resemble real-world settings as closely as possible. The third dataset (Test set 3) was constructed by randomly sampling images obtained using a photon-counting CT (PCCT) scanner (acquisition protocol described in Supplementary material “Test set 3 (PCCT) protocol”). The fourth dataset (Test set 4) comprised images obtained using lower radiation exposure, approximately 25% of the dose applied using the protocol of Dataset 1. The dose reduction was measured using CT Dose Index (CTDvol) values (these examinations were conducted following a request for suspected nephrolithiasis).

**Fig. 1:**
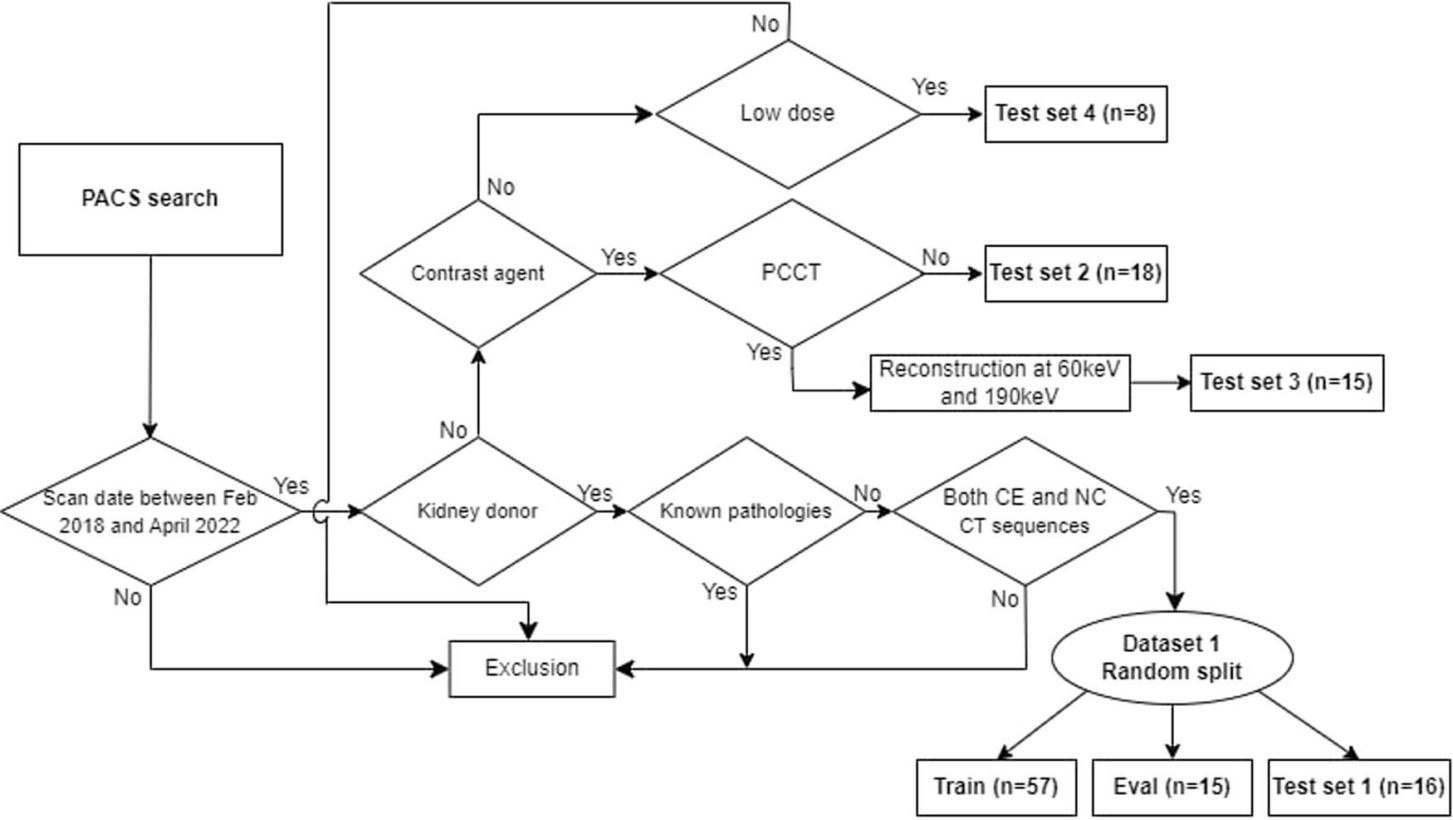
Subject inclusion process. PACS, Picture archiving and communications system; CE, Contrast-enhanced; NC, Noncontrast; CT, Computed tomography; PCCT, Photon-counting CT

The inclusion process is depicted in Fig. 1. Test sets 2, 3, and 4 were used only for testing purposes. The ground truth labels for this study were constructed using the SegmentEditor module of 3D Slicer [28]. Manual delineation of both the left and right kidneys was performed, excluding the renal sinus fat. The annotation process of Dataset 1 was performed by a medical student (radiology intern with 2 years of experience ('sH.R.)) under the guideline and inspection of an expert radiologist with 15 years of experience (D.W.L.). Test set 2 annotation was performed by this expert radiologist, in order to validate the model performance against the labels of an experienced clinician.

**Fig. 2:**
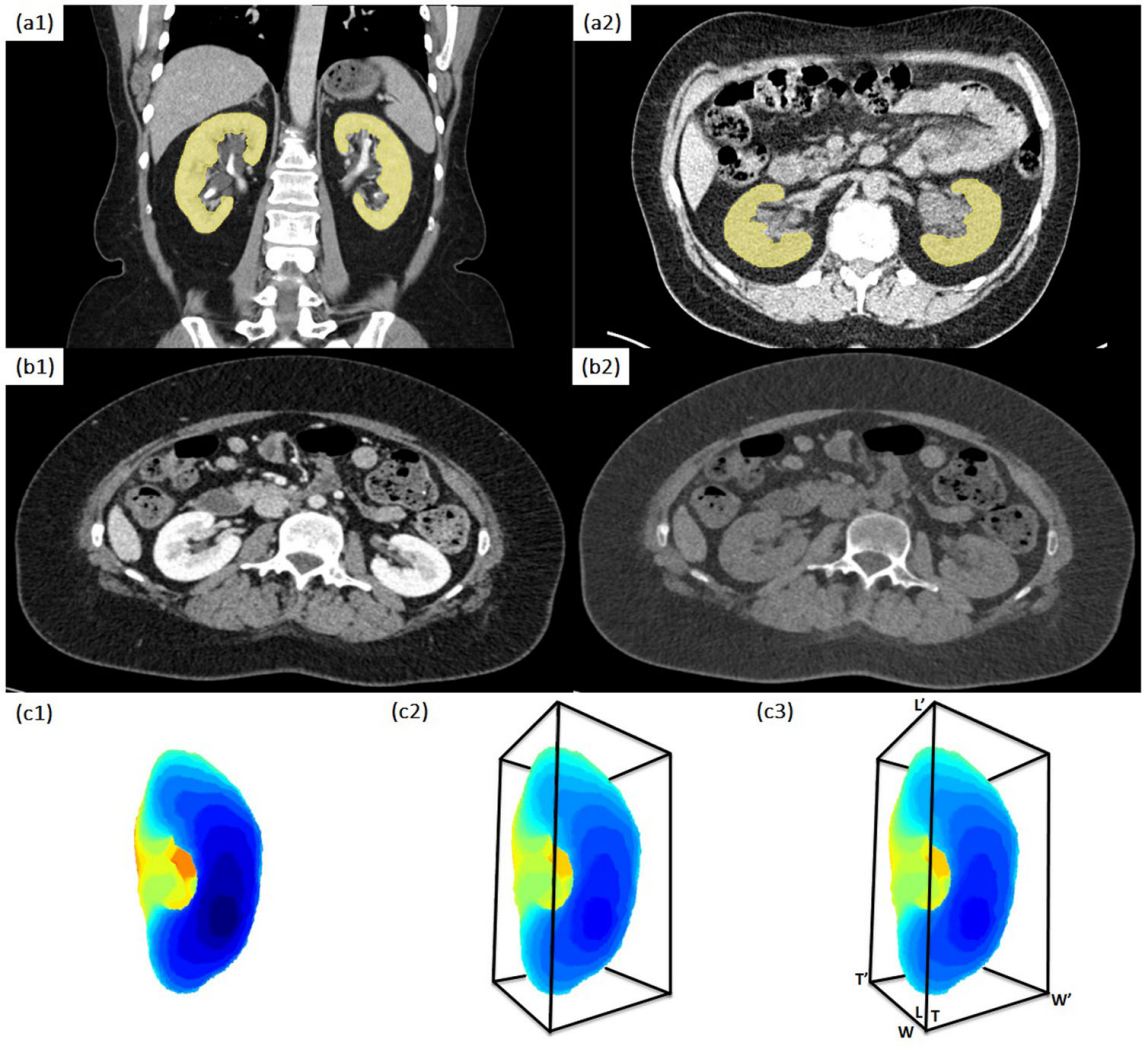
**a1**, **a2** Contrast-enhanced (left) and noncontrast (right) computed tomography scans and ground truth segmentations. The renal sinus fat has been excluded during the manual delineation process. **b1**, **b2** Photon-counting computed tomography image reconstructed at 60 keV (left) and 190 keV (right). The 60-keV image resembles an image acquired using contrast agent, while the 190 keV reconstruction resembles an image without contrast. **c1** Three-dimensional point cloud constructed from the segmentation mask. **c2** Bounding box. **c3** Renal axes calculation

Test set 3 comprised virtual monoenergetic images obtained using a PCCT scanner, reconstructed at 60 and 190 keV. The low energy (60 keV) reconstructions visually resemble CE images, while the high energy ones (190 keV) resemble NC images respectively. The annotation of Test set 3 was performed by the same expert radiologist who performed Test set 2 annotation. Test set 4 was composed of scans obtained using lower x-ray doses compared to that of Dataset 1 in order to assess the model performance when CT images are obtained at low x-ray doses. The annotation of Test set 4 was performed by the same expert radiologist. Examples of manual segmentations for CE and NC CT images of Dataset 1 are shown in Fig. 2a1, a2. PCCT images, reconstructed at 60 and 190 keV, are depicted in Fig. 2b1, b2. Data collection involved the measurement of kidney volumes and multidimensional axes using the generated manual labels, as summarized in Table 1, along with relevant dataset characteristics.

**Table 1:** Datasets characteristics

Parameter	Dataset 1 CE	Dataset 1 NC	Test set 2	Test set 3 PCCT		Test set 4
Number of subjects	88	88	18	15		8
Females/males	49/39	49/39	13/5	9/6		3/5
kVp	[100, 100] (100)	[100, 120] (100)	[82.5, 100] (100)	(120)		[100, 125] (110)
Kidney density (HU)	[141, 204] (171)	[18, 38] (28)	[148, 219] (182)	60 keV	[176, 247] (214)	[17, 48] (33)
				190 keV	[24, 45] (35)	
CTDIvol (mGy)	[8.13, 10.51] (8.16)	[8.73, 9.81] (9.41)	[7.08, 9.30] (8.77)	[3.68, 6.73] (5.22)		[1.58, 2.15] (1.83)
Scanner						
Manufacturer	Siemens	Siemens	Siemens	Siemens		Siemens
Model	SOMATOM Definition Flash, Force	SOMATOM Definition Flash, Force	SOMATOM Definition Flash, Force, Definition Edge	NAEOTOM Alpha		SOMATOM Definition Flash, Force
Image size	(512, 512, 101)	(512, 512, 48)	(512, 512, 101)	(513, 512, 677)		(512, 512, 131)
Pixel spacing (mm)	(0.86, 0.86)	(0.69, 0.69)	(0.83, 0.82)	(0.82, 0.82)		(0.69, 0.69)
Slice thickness (mm)	(3)	(5)	(3)	(0.7)		(3)
Age (years)	[36, 59.2] (49)	[36, 59.2] (49)	[39, 56] (47)	[54.5, 70] (60)		[31.5, 45.2] (40)
Volume (mL)	[121.39, 160.43] (139.83)	[125.81, 159.26] (140.87)	[118.1, 151.46] (135.97)	[106.27, 148.97] (118)		[137.79, 181.49] (146.96)
Length (mm)	[107.36, 120.29] (113.96)	[109.25, 121.82] (115.80)	[106.32, 119.2] (112.81)	[100.16, 114.14] (111.78)		[112.66, 124.79] (117.27)
Width (mm)	[63.21, 69.18] (66.28)	[63.13, 69.67] (66.58)	[62.79, 69.52] (65.97)	[57.46, 66.16] (61.52)		[66.97, 70.61] (69.33)
Thickness (mm)	[44.56, 53.43] (49.18)	[45.47, 53.31] (48.89)	[46.26, 52.13] (48.53)	[43.72, 48.81] (46.52)		[47.93, 54.42] (51.57)

Values in brackets represent interquartile ranges; values in parentheses represent medians. Kidney density values are given in HU by retrospective analysis using manual annotations

*CE* Contrast-enhanced, *NC* Noncontrast, *PCCT* Photon-counting computed tomography

### Preprocessing

The studied cohort of Dataset 1 was randomly split into training, validation, and test sets (ratio of 0.65, 0.175, and 0.175, respectively). CE and NC images were used to train two separate networks. To facilitate the network training process, NC images underwent resampling using trilinear interpolation to achieve isotropic spacing of 1.5 mm^3^, resulting in a median image size of 236 × 236 × 156. CE images were not resampled but rather used at their original resolutions, as their spacing had much less variation. NC and CE images were truncated with a window level/width of 65/170 HU and 200/300 HU, respectively, and then normalized to [0, 1] using min-max normalization. The choice of this windowing setting was based on the kidney HU distribution of the NC and CE images. Kidney labels were transformed to one-hot encoded representations for both CE and NC cases using three represented classes: background, right kidney, and left kidney. Data augmentation techniques were used to artificially extend the training cohort, including random patch extraction, random flipping in *x*, *y*, and *z* axes, random intensity shifting (offset < 0.1), and addition of Gaussian noise (0 ± 0.05, mean ± standard deviation).

### Network implementation and training

**Fig. 3:**
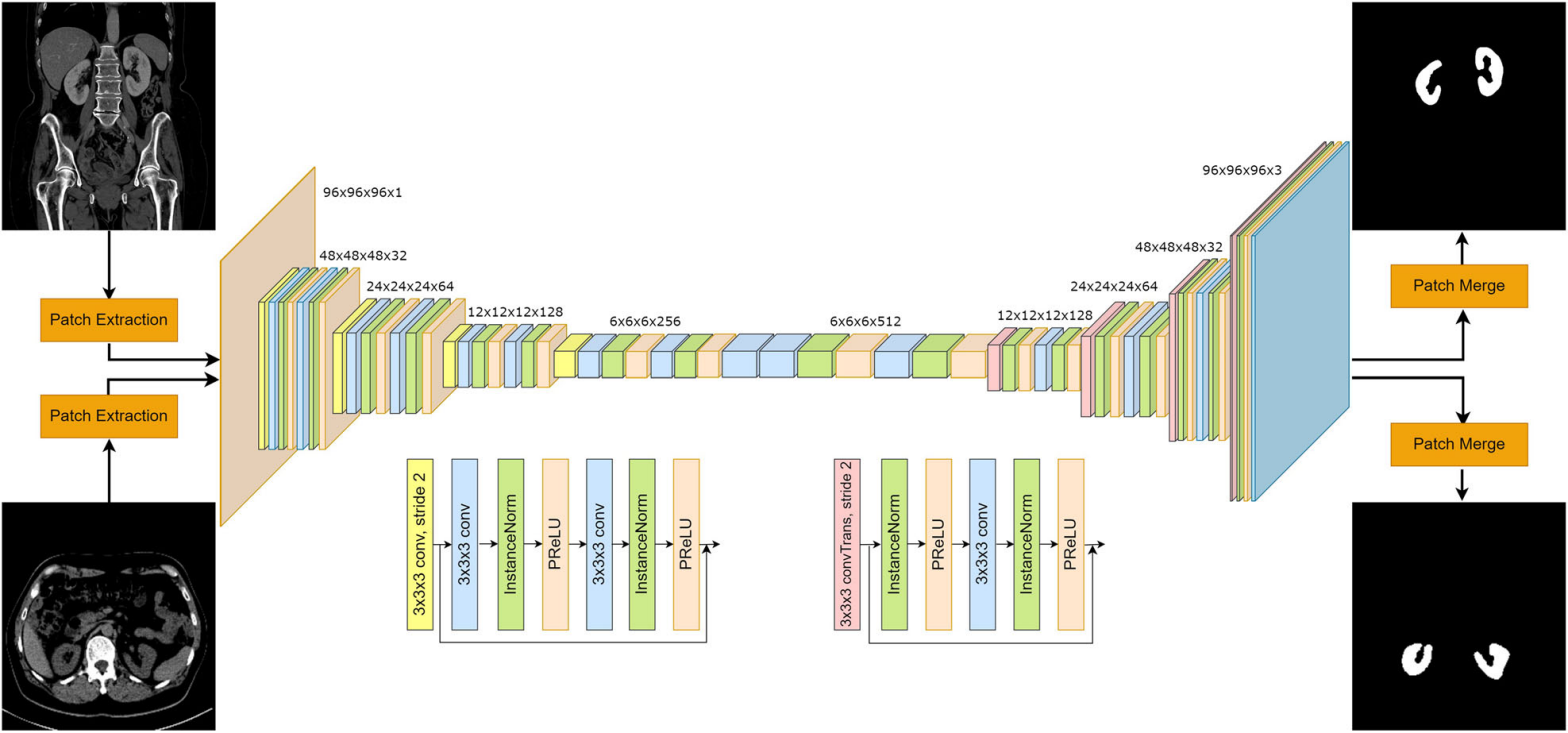
UNet architecture. The input CT patch is downsampled 4 times by a factor of two in every encoding layer while the number of channels is doubled. The reverse process takes place in the decoder path. Both the encoder and decoder blocks are based on residual units

Each of the CE and NC image sets was used to train a 3D UNet [29] convolutional neural network for the automated segmentation of both the left and right kidneys. The architecture of both networks was identical, as depicted in Fig. 3. The corresponding blocks of the encoder and decoder made use of residual connections [30], in order to facilitate the network training and minimize vanishing gradients that would halt the convergence. We used Adam [31] to optimize the parameters of each model with a constant learning rate of 3 × 10^−^^4^, and Dice loss [32] as objective criterion. Furthermore, to avoid overfitting, a dropout rate of 0.3 was used in both networks. The batch size was 8 and each sample in the batch consisted of a random cropped patch from the initial CT, ${Pi\; \in } \, {{\mathbb{R}}}^{96x96x96}{i}=\{1,\ldots ,8\}$. For each of the training experiments, we made use of 1 NVIDIA GeForce RTX 3090 with 24 GB of memory, and all models were trained for 1500 epochs. The network architecture was implemented using PyTorch v1.10.0 (https://pytorch.org/) and MONAI v1.0.1 (https://monai.io/), while the training was implemented using CUDA 11.3. The CECT model took approximately 9 h to train while the NCCT took about 4 h, as NC images were smaller due to the resampling process.

### Inference and post-processing

During model inference, 24 overlapping patches, ${Pi}\in {{\mathbb{R}}}^{96x96x96},{i}=1,\ldots ,24$, for every scan are passed through the network in a sliding window setting, using a patch overlap of 0.5 and averaging the predictions of overlapping pixels. At the model output, the argmax function was applied to the raw logits, converting it to a one-hot encoded representation of three classes (background, right kidney, left kidney). We then perform connected component analysis to each of the kidney classes, keeping only the largest segment (*i.e*., the kidney). NC images are also upsampled to the original resolution using trilinear interpolation. As this interpolation process assigns nonzero values to neighboring voxels of the kidney mask, an appropriate threshold needs to be set to avoid under/over-estimation of the kidney. To this end, we predict the validation set cases using different threshold settings ${Ti}\in [0,\,1]$, and choose the most suitable one based on the segmentation performance.

### Quantitative measurements

Once both kidneys had been segmented, volumetric measurements were straightforward to derive by multiplying the number of foreground voxels by the initial spacing, available in the DICOM header. Nevertheless, to calculate the three kidney axes (length, width, and thickness) requires additional processing. To this end, each kidney is first interpolated to an isotropic spacing of 1 mm^3^, and the main kidney axes are obtained by principal component analysis of the 3D point cloud of kidney voxels. The minimal bounding box oriented along these axes and enclosing the kidney is then determined and its extents are measured from the center along each axis direction. In this way, the three axes are defined as the longest distances inside the kidney in the following directions: length (inferior to superior), width (medial to lateral), and thickness (ventral to dorsal). Figure 2b1–b3 illustrates the process of calculating the renal axes. The kidney length equals the distance LL’, while the width is equal to WW’, and the thickness equals TT’. The kidney bounding box was calculated using Open3D v0.15 (https://www.open3d.org/).

### Performance benchmarking using TotalSegmentator

In order to obtain a baseline performance for the test subset of Dataset 1, we used a publicly available model, namely TotalSegmentator [33], to predict both the CE and NC cases. The model was directly downloaded from its GitHub repository (https://github.com/wasserth/TotalSegmentator). To allow for a fair comparison, we used the 3 mm resolution model to segment the CE cases, while for the NC dataset, we used the higher resolution model of 1.5 mm.

### Volumetric measurements based on the ellipsoid model

Manual delineations for both the left and right kidneys were used to calculate renal volumes of Test set 1 based on the ellipsoid model. Once the kidney axes have been calculated using the aforementioned bounding box approach, the kidney volume can be estimated using the formula below:

$${Ellipsoid\; volume}=\,\frac{\pi }{6}x \, {length} \, x \, {width} \, x \, {thickness}$$

### Statistical analysis

The accuracy of the automated measurements calculated based on the segmentations of the deep learning models was determined by comparison against the ground truth measurements from manual delineation. Statistical analysis was performed using Python v3.9.18. The performance of our deep learning-based networks is based on their ability to measure accurately the renal volumes and axes. This is first evaluated in terms of the Dice similarity coefficient (DSC), a measure of spatial overlap between segmentations where a DSC of 1 implies perfect overlap while a DSC of 0 means complete mismatch. The mean percentage volume error and the mean absolute error were incorporated to compare renal volume and axes measurements, defined as:

$${Mean\; percentage\; volume\; error} \, \left( \% \right)=\,\frac{\left|{vo}{l}_{{man}}-{vo}{l}_{{pred}}\right|}{{vo}{l}_{{man}}}x100$$

$${Mean\; absolute\; error}=\left|{x}_{{man}} \! -{x}_{{pred}}\right|, x \! \in \! [{length},{width},{thickness}]$$

where ${man}$ and ${pred}$ denote manual and predicted values, respectively. Intraclass correlation coefficient (ICC) estimates the reliability of automated *versus* manual measurements. ICC values were calculated using the pingouin Python package v0.5.3 (https://pingouin-stats.org/build/html/index.html) based on a single-rater, consistency measurements, 2-way mixed effects model. ICC values can be interpreted as follows [34]: poor (ICC < 0.50); moderate (0.50 ≤ ICC < 0.75); good (0.75 ≤ ICC < 0.90); and excellent (ICC ≥ 0.90); 95% confidence intervals (CIs) were also calculated. Furthermore, Bland-Altman analysis was used to demonstrate the differences in volumetric agreement between the models and the ground truth.

## Results

### Manual intraobserver variability

**Fig. 4:**
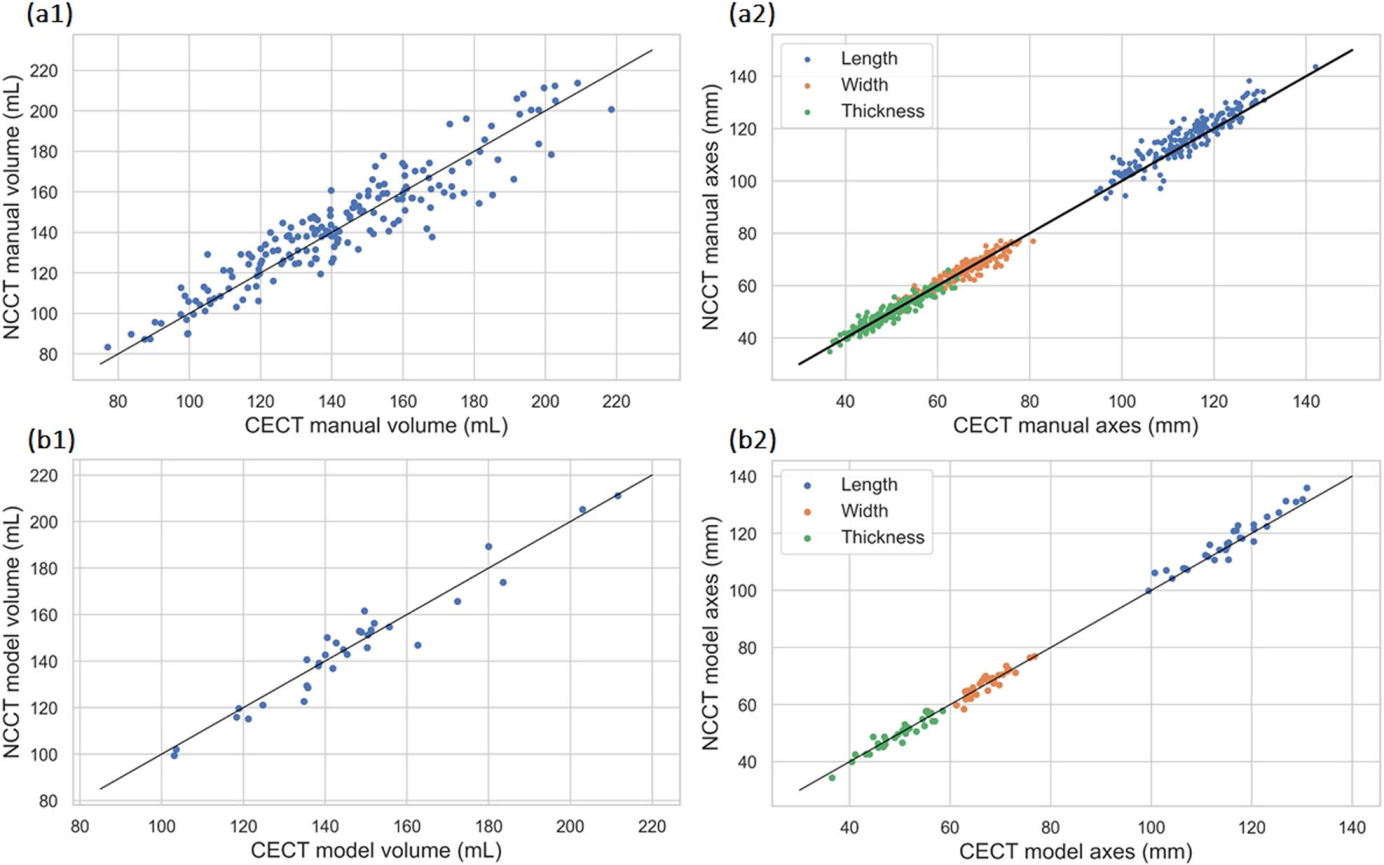
**a1**, **a2** Correlation of measured renal volumes and axes in CECT and NCCT images by the manual annotator (entire Dataset 1, *n* = 88). **b1**, **b2** Correlation of measured renal volumes and axes in CECT and NCCT images by CECT and NCCT models (Test set 1, *n* = 16). CECT, Contrast-enhanced computed tomography; NCCT, Noncontrast computed tomography

The intraobserver variability between the CECT and NCCT ground truth measurements was evaluated using the ICC. For renal volume, the ICC reached 0.93 (95% CI: [0.91, 0.95]) (*p* < 0.001), demonstrating a high level of agreement between the observer’s measurements in CECT and NCCT. Similarly, for kidney length, width, and thickness, the ICC values were 0.92 (95% CI: [0.90, 0.94]) (*p* < 0.001), 0.93 (95% CI: [0.91, 0.95]) (*p* < 0.001), and 0.94 (95% CI: [0.92, 0.96]) (*p* < 0.001) respectively. Volume and axes correlation plots for the entire Dataset 1 (*n* = 88) are depicted in Fig. 4a1, a2.

### CECT model evaluated against CECT ground truth of Test set 1

**Fig. 5:**
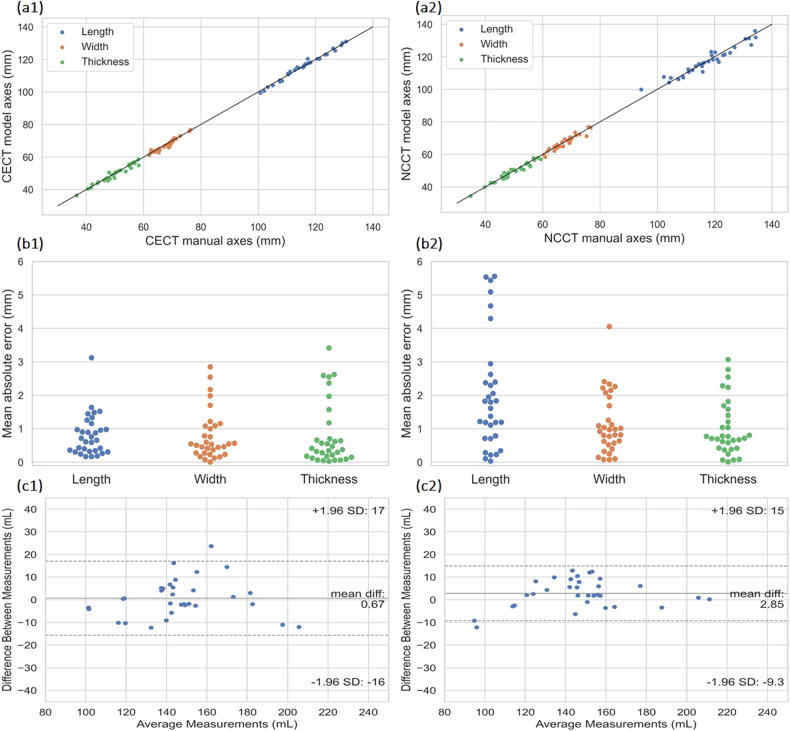
Test set 1 (*n* = 16) **a1**, **a2** Correlation in axes measurements between CECT model *versus* manual and NCCT model *versus* manual. **b1**, **b2** Mean absolute error in axes measurements between CECT model *versus* manual and NCCT model *versus* manual. **c1**, **c2** Bland-Altman analysis of differences in kidney volume between CECT model *versus* manual and NCCT model *versus* manual. CECT, Contrast-enhanced computed tomography; NCCT, Noncontrast computed tomography

Table 2 demonstrates the CECT model performance, in Test set 1 (*n* = 16), in estimating the renal volume, length, width, and thickness, and the average model inference time. The ICCs substantiated the reliability of this method, which suggests that the model reliability is good to excellent when estimating renal volume, while for the rest of the renal parameters the reliability was excellent. Figure 5a1, b1 demonstrates the correlation and error for measured axes compared to the manual ground truth, while the Bland-Altman analysis for the volumetric measurements between the model and the manual annotator demonstrated excellent agreement (Fig. 5c1). Example CECT images of Test set 1, along with their corresponding manual segmentations and model predictions, can be found in Fig. S1a1, a2 and Fig. S2a1, a2 in the Supplementary material.

**Table 2:** Automated *versus* manual measurements

Parameter	Test set 1 CE	Test set 1 NC	Test set 2	Test set 3 60 keV	Test set 3 190 keV	Test set 4
Dice similarity coefficient (mean ± SD)	0.95 ± 0.01 ([0.94, 0.95])	0.94 ± 0.01 ([0.93, 0.94])	0.94 ± 0.01 ([0.94, 0.96])	0.92 ± 0.03 ([0.89, 0.94])	0.92 ± 0.03 ([0.91, 0.94])	0.93 ± 0.02 ([0.92, 0.94])
Mean percentage volume error (mean ± SD)						
Renal volume (mL)	4.15 ± 2.92%	4.06 ± 3.18%	3.85 ± 3.62%	6.16 ± 5.58%	5.65 ± 6.04%	7.16 ± 4.98%
Mean absolute error (mean ± SD)						
Length (mm)	0.80 ± 0.61	2.02 ± 1.69	1.51 ± 1.70	1.74 ± 1.26	1.90 ± 1.72	1.14 ± 1.12
Width (mm)	0.78 ± 0.73	1.13 ± 0.89	1.14 ± 0.91	1.61 ± 1.50	0.91 ± 0.64	1.14 ± 0.74
Thickness (mm)	0.80 ± 0.94	1.01 ± 0.82	0.94 ± 1.12	1.84 ± 1.12	1.28 ± 2.56	0.63 ± 0.49
Intraclass correlation coefficient						
Volume	0.94 ([0.88, 0.97], *p* < 0.001)	0.97 ([0.94, 0.99], *p* < 0.001)	0.95 ([0.91, 0.98], *p* < 0.001)	0.95 ([0.91, 0.98], *p* < 0.001)	0.96 ([0.94, 0.99], *p* < 0.001)	0.95 ([0.88, 0.98], *p* < 0.001)
Length	0.99 ([0.98, 1.0], *p* < 0.001)	0.95 ([0.92, 0.98], *p* < 0.001)	0.98 ([0.97, 0.99], *p* < 0.001)	0.99 ([0.98, 1.00], *p* < 0.001)	0.96 ([0.93, 0.98], *p* < 0.001)	0.98 ([0.95, 0.99], *p* < 0.001)
Width	0.97 ([0.95, 0.99], *p* < 0.001)	0.95 ([0.91, 0.98], *p* < 0.001)	0.95 ([0.91, 0.98], *p* < 0.001)	0.97 ([0.94, 0.99], *p* < 0.001)	0.90 ([0.82, 0.96], *p* < 0.001)	0.91 ([0.77, 0.97], *p* < 0.001)
Thickness	0.97 ([0.95, 0.99], *p* < 0.001)	0.97 ([0.95, 0.99], *p* < 0.001)	0.97 ([0.94, 0.99], *p* < 0.001)	0.94 ([0.89, 0.97], *p* < 0.001)	0.98 ([0.98, 0.99], *p* < 0.001)	0.98 ([0.96, 1.0], *p* < 0.001)
Inference time (s) (mean ± SD)	7.9 ± 1.3	4.6 ± 0.6	8.7 ± 1.6	35.6 ± 3.9	34.4 ± 3.7	18.2 ± 11.1

Values in brackets represent 95% confidence intervals. The inference time is that taken by the model to predict a CT scan

*SD* Standard deviation

### NCCT model comparison with NCCT ground truth of Test set 1

Renal volume estimation error and ICC values for volume, length, width, and thickness measurements of the NCCT model are depicted in Table 2, along with average model inference time, akin to the performance observed in the CECT scenario. ICC values indicate reliability which is on par with the CECT model for the renal axes measurements, while in the case of renal volume, the NCCT model demonstrates excellent reliability, based on the measured 95% CI. Figure 5a2, b2 demonstrate the correlation and measured error in axes calculation between the model and the ground truth, while the Bland-Altman analysis for the volumetric agreement between the two methods is depicted in Fig. 5c2. Example NCCT images from Test set 1, along with their corresponding manual segmentations and model predictions can be found in Fig. S1b1, b2 and Fig. S2b1, b2 in the Supplementary material.

### CECT *versus* NCCT model agreement for Test set 1

A comparison was conducted between the CECT and NCCT segmentation models. The ICCs for renal volume, length, width, and thickness were consistently high, with values of 0.96 (95% CI: [0.94, 0.99]) (*p* < 0.001), 0.96 (95% CI: [0.9, 0.98]) (*p* < 0.001), 0.92 (95% CI: [0.85, 0.96]) (*p* < 0.001), and 0.95 (95% CI: [0.90, 0.98]) (*p* < 0.001), respectively. Figure 4b1, b2 demonstrates the level of agreement between the models.

### TotalSegmentator benchmark for Test set 1

The TotalSegmentator model was used to obtain a baseline performance on Test set 1. The 3-mm resolution model, segmenting CECT images, achieved a DSC of 0.92 (95% CI: [0.91, 0.92]), and our CECT model had a DSC of 0.95 (*p* < 0.001). The average volume error was 7% mL, and the ICC for renal volume was 0.94 (95% CI: [0.84, 0.98]) (*p* < 0.001), indicating good to excellent reliability in renal volume estimation. The 1.5-mm resolution model, segmenting NCCT images, achieved a DSC of 0.93 (95% CI: [0.91, 0.94]), and our NCCT model achieved a DSC of 0.95 (*p* < 0.001). The average volume error was 3% mL, and the ICC value for renal volume was 0.98 (95% CI: [0.96, 1]) (*p* < 0.001), demonstrating excellent reliability. The average prediction time for the CECT images was 8.9 ± 1.0 s, while for the NCCT was 46.3 ± 6.2 s (mean ± standard deviation).

### Comparison with the ellipsoid model

**Fig. 6:**
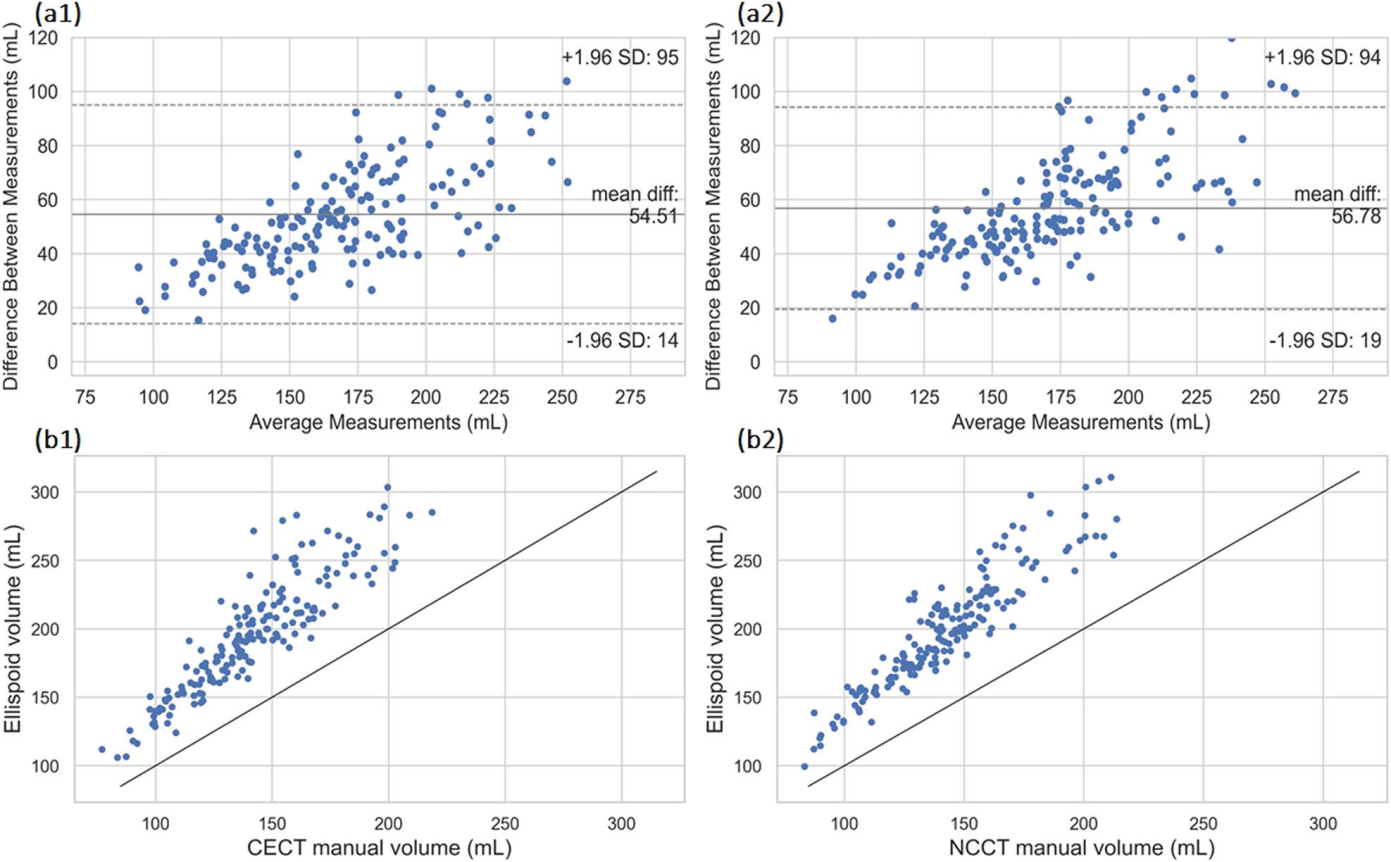
Dataset 1 (*n* = 88) **a1**, **a2** Bland-Altman analysis between ellipsoid model *versus* manual for CE and NC cases. **b1**, **b2** Correlation of measured volumes between ellipsoid model *versus* manual for CE and NC cases. CECT, Contrast-enhanced computed tomography; NCCT, Noncontrast computed tomography

An additional comparative analysis was conducted between the CECT and NCCT ground truth labels and the ellipsoid formula for renal volume calculation on the entire dataset (*n* = 88). The ellipsoid model exhibited a mean volume measurement error of 38 ± 12% mL and 39 ± 10% mL for the CE and NC images (mean ± standard deviation) respectively, indicating a considerable discrepancy in volume estimation when compared to the manual segmentations, as shown in Fig. 6. The ICC value for renal volume estimation was 0.83 (95% CI 0.78–0.87, *p* < 0.001) and 0.85 (95% CI 0.81–0.89, *p* < 0.001) for CE and NC cases respectively, implying good reliability but significantly inferior compared to that of the CECT and NCCT models.

### CECT model validation on Test set 2

**Fig. 7:**
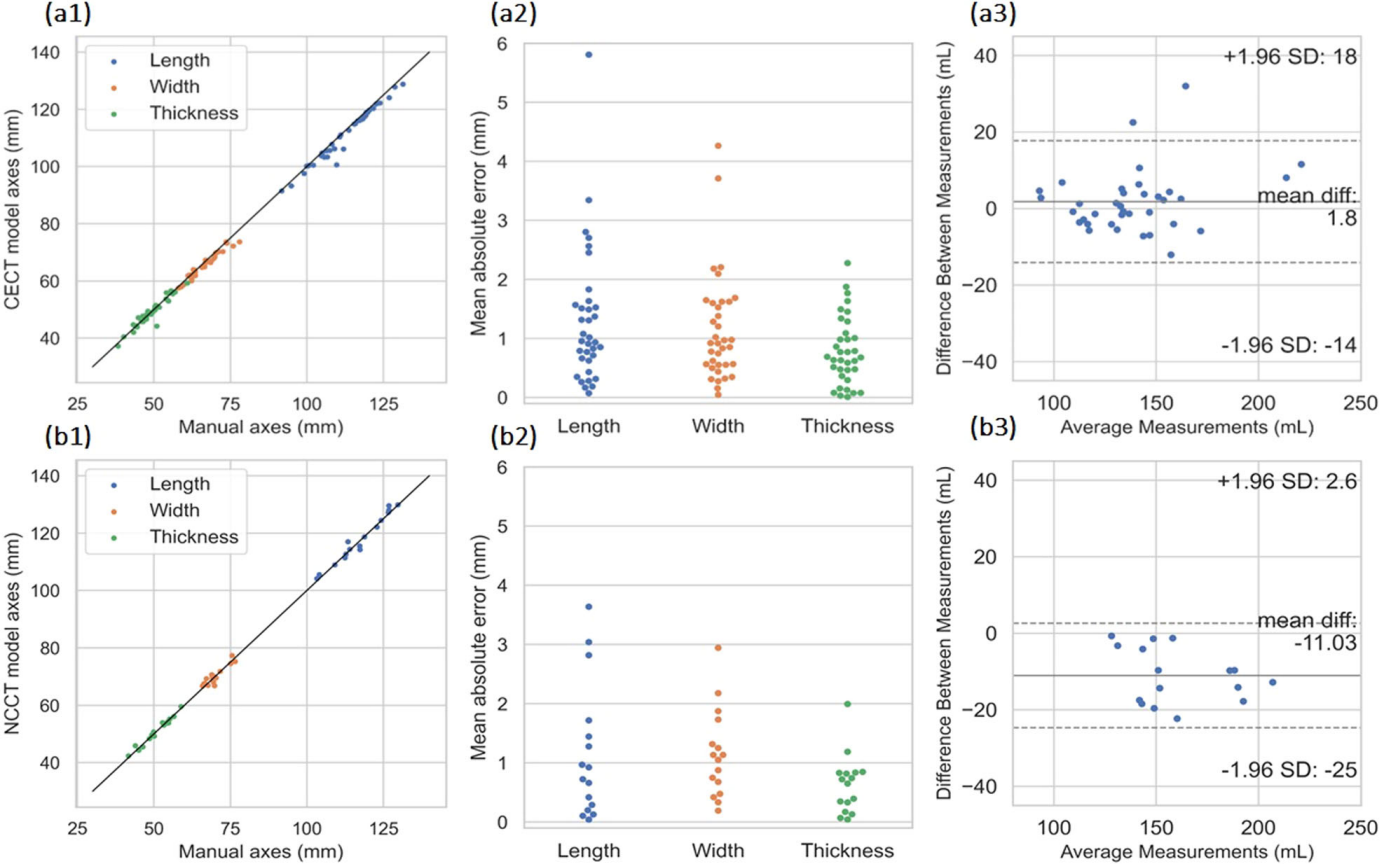
Test set 2 (*n* = 18). **a1** Correlation in axes measurements between CECT model *versus* manual; **a2** mean absolute error in axes measurements between CECT model *versus* manual; **a3** Bland-Altman analysis of differences in kidney volume between CECT model *versus* manual. Test set 4 (*n* = 8). **b1** Correlation in axes measurements between NCCT model *versus* manual; **b2** mean absolute error in axes measurements between NCCT model *versus* manual; **b3** Bland-Altman analysis of differences in kidney volume between NCCT model *versus* manual. CECT, Contrast-enhanced computed tomography; NCCT, Noncontrast computed tomography

The CECT model was evaluated against a separate test set constructed by the expert radiologist. The average manual annotation time per case was measured as 25.8 ± 3.4 min (mean ± standard deviation). The model segmentation performance demonstrated a high DSC, comparable with the performance of Test set 1. Renal volume estimation error and ICC values for renal length, width, and thickness were akin to the ones obtained in Test set 1, as depicted in Table 2. Figure 7a1, a2 demonstrates the correlation and errors of renal axes predicted by the model, compared to the expert radiologist. Bland-Altman analysis of renal volume estimation is depicted in Fig. 7a3.

### CECT and NCCT model validation on PCCT Test set 3

**Fig. 8:**
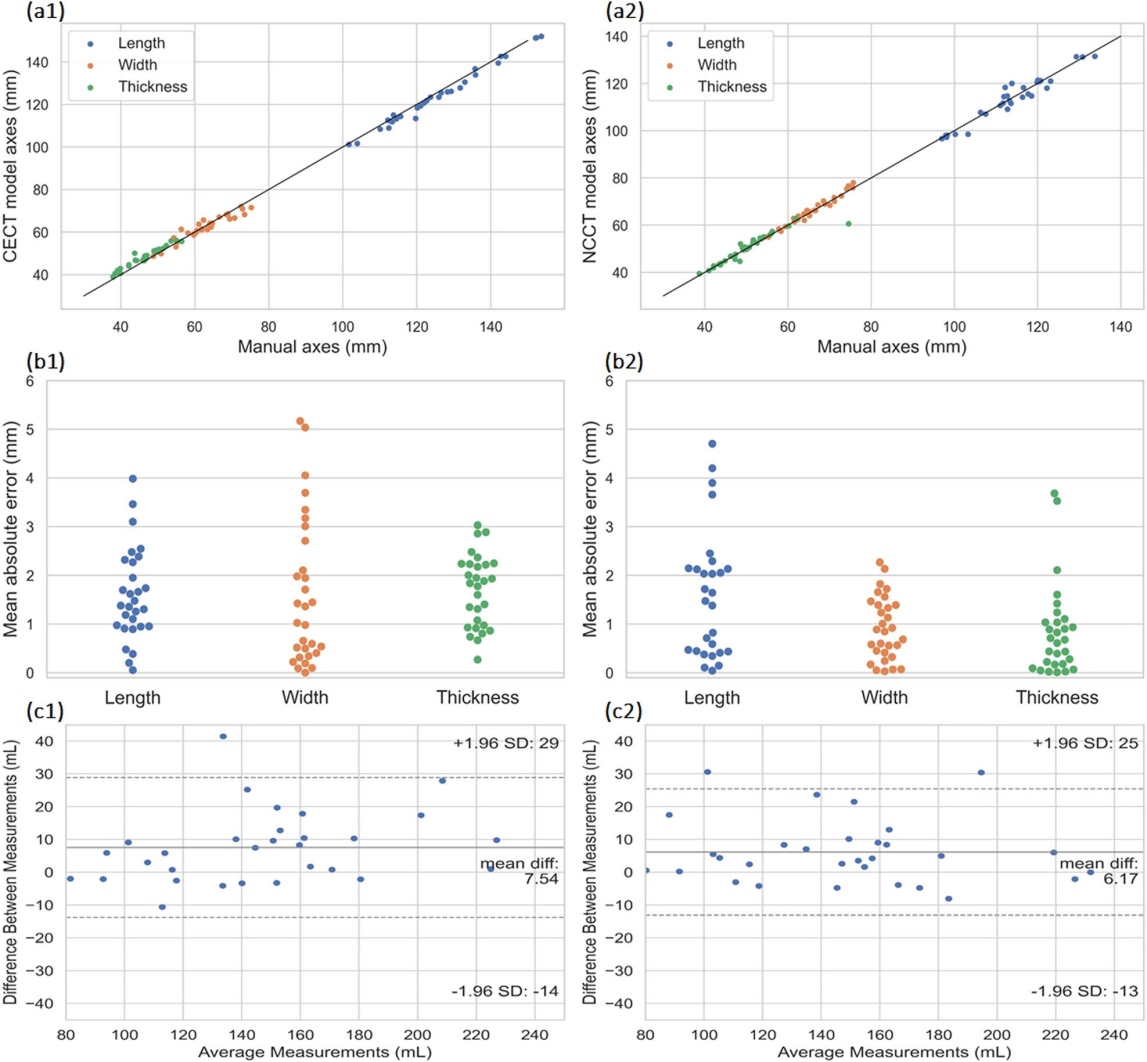
Test set 3 (PCCT, *n* = 15). **a1**, **a2** Correlation in axes measurements between CECT model *versus* manual and NCCT model *versus* manual. **b1**, **b2** Mean absolute error in axes measurements between CECT model *versus* manual and NCCT model *versus* manual. **c1**, **c2** Bland-Altman analysis of differences in kidney volume between CECT model *versus* manual and NCCT model *versus* manual. CECT, Contrast-enhanced computed tomography. NCCT, Noncontrast computed tomography; PCCT, Photon-counting computed tomography

The CECT and NCCT models we evaluated against an external dataset of PCCT images reconstructed at 60 keV and 190 keV, respectively. Images reconstructed at 60 keV resembled those acquired with contrast agent in a non-PCCT scanner, while the ones reconstructed at 190 keV resembled images acquired using a NC protocol. The models demonstrated high DSC and ICC values in estimating renal quantitative measurements, supported by 95% CI, reported in Table 2. Figure 8a1, b2 illustrates correlation and error diagrams of the CECT and NCCT models compared to the manual measurements, while Fig. 8c1, c2 demonstrates Bland-Altman plots for the volumetric agreement between the models and the ground truth volumes.

### NCCT model validation on Test set 4

The NCCT was evaluated against a test set obtained using significantly lower dose levels compared to the training cohort. The segmentation performance demonstrated high DSC, comparable to that of Test set 1. Estimation of renal volume and ICC values for renal length, width, and thickness did not decrease significantly compared to Test set 1, depicted in Table 2. Renal axes measurement correlation and errors, predicted by the NCCT model, compared to the ground truth from the expert radiologist are demonstrated in Fig. 7b1, b2. Figure 7b3 illustrates the Bland-Altman analysis of renal volume estimation.

## Discussion

In this study, we developed two 3D UNet segmentation networks for kidney segmentation based on CE and NC CT images, to help radiologists derive quantitative renal measurements.

Intraobserver variability between the CE and NC ground truth emphasized the consistent and reliable nature of the manual annotation process, providing a solid foundation for the subsequent evaluation of automated methods.

Evaluation of the CECT model compared to the ground truth revealed promising results. The DSC demonstrated a high level of agreement, and the model exhibited accurate renal volume estimations with a limited volume error. The ICCs (ICC, 95% CI) for volume (0.94, 0.88–0.97), length (0.99, 0.98–1.0), width (0.97, 0.95–0.99), and thickness (0.97, 0.95–0.99) demonstrate good-to-excellent reliability in deriving quantitative renal measurements. Validation of the CECT model on a separate test set, annotated by an expert radiologist, demonstrates excellent reliability in estimating renal volume (0.95, 0.91–0.98), length (0.98, 0.97–0.99), width (0.95, 0.91–0.98), and thickness (0.97, 0.94–0.99). Furthermore, the CECT model was able to generalize its predictions in an external test set obtained using PCCT scanner images reconstructed at 60 keV. The model preserved high DSC and excellent reliability for measurements of renal volume (0.95, 0.91–0.98), length (0.99, 0.98–1), width (0.97, 0.94–0.99), and thickness (0.94, 0.89–0.97).

Similar to the CECT case, the evaluation of the NCCT segmentation model against the NCCT ground truth demonstrated robust performance. The measured DSC highlights the model’s ability to delineate renal structures even for NC scans. The ICCs for volume (0.97, 0.94–0.99), length (0.95, 0.92–0.98), width (0.95, 0.91–0.98), and thickness (0.97, 0.95–0.99) manifest the NCCT model excellent reliability in measuring renal parameters. Additional validation of the NCCT model with images of a PCCT scanner reconstructed at 190 keV highlights its robustness. The ICCs in estimating volume (0.96, 0.94–0.99), length (0.96, 0.93–0.98), width (0.90, 0.82–0.96) and thickness (0.98, 0.98–0.99) manifest the NCCT model ability to generalize well in new datasets, acquired using different CT scanners. The validation of the NCCT model in a separate test set (Test set 4), obtained using lower dose levels compared to those of the training cohort (~ 25% of the dose) demonstrates good-to-excellent reliability in renal volume estimation (0.95, 0.88–0.98), and measurement of renal length (0.98, 0.95–0.99), width (0.91, 0.77–0.97), and thickness (0.98, 0.96–1.0).

These findings indicate excellent agreement between the automated methods and the manual reference standard. Our analysis highlights the remarkable consistency and reliability of both CECT and NCCT, emphasizing their comparable performance in capturing key anatomical features. Such interchangeability adds versatility to their application, suggesting that both approaches can be effectively employed conversely depending on the imaging modality or clinical requirements, offering flexibility and robust performance in renal imaging analyses. Our investigation also underpins the effectiveness of automated measurement methods in assisting radiologists with accurate and reproducible quantification of renal clinical descriptors. The widespread ellipsoid formula tends to overestimate renal volumes, especially when the kidney size becomes large, demonstrating low reliability and extensive large-volume measurement errors for both CE and NC cases. Moreover, validation of an external, freely available and publicly recognized segmentation model (TotalSegmentator) enabled to establish a performance benchmark for the study dataset. Our methods performed slightly better than TotalSegmentator in terms of DSC both for the CE (0.95 and 0.92 respectively, *p* < 0.001) and the NC (0.95 and 0.93 respectively, *p* < 0.001) cases, while being faster at segmenting the structure of interest.

Although many studies have demonstrated excellent performance in kidney segmentation, most of them did not address the issue of clinical evaluation of renal axes, limiting their applicability. Methods developed using part of the KiTS challenge dataset, although accurate and developed using a diverse and rich dataset, suffer from the inclusion of the renal sinus fat, which does not contribute as functional tissue. Additionally, the challenge is based on CE images only, while in clinical settings, the use of intravenous contrast agent may need to be avoided. Excluding the non-functional tissue, Milecki et al [35] reported a DSC of 0.89 ± 0.31 when segmenting kidneys without the sinus fat in MRI sequences of 32 patients subject to kidney transplantation. Korfiatis et al [36] segmented renal cortex and medulla separately in arterial phase CT, achieving a DSC of 0.94 ± 0.01 for the cortex and 0.90 ± 0.03 for the medulla using an extended dataset from a single institution (*n* = 1,930) and two additional external test sets (*n* = 1,226). Valente et al [37] used two-dimensional ultrasound to segment the kidneys and reported a DSC of 0.86 ± 0.11 using a cohort of size similar to the one in our study (*n* = 66). The large variance reported in their results attests to the inferiority of ultrasound-based measurements compared to CT. Muller et al [38] used 210 low-dose NCCT images with manual segmentations to develop a network and tested it against 22 semiautomated volume estimates from radiologists, reporting DSCs of 0.91 for the right and 0.86 for the left kidney. A limitation of their work is the use of a two-dimensional convolutional neural network, which can prolong the segmentation process considerably compared to the 3D counterpart and can lead to suboptimal results in terms of segmentation performance. Oliveira et al [39] used only five CT images to test an active contour model, segmenting the kidney and the renal collecting system separately, reporting DSCs of 0.92 ± 0.01 and 0.63 ± 0.10, respectively. However, a user must manually provide seed points for the entire model to start the segmentation process.

Measuring renal axes is a topic that has been studied extensively using manual approaches [11, 19, 40, 41]. Knowledge about the overall renal morphology and anatomical characteristics of renal axes can facilitate surgical planning (*e.g*., in kidney transplantation procedures), and further assist in post-transplantation renal assessment where the remaining kidney is expected to increase in size. Pre- and post-transplantation renal axis measurements can help the clinicians to identify how the remaining kidney developed, *i.e*., increase in length and/or width. Furthermore, measuring renal axes is commonly performed in ultrasound [1, 2, 4] because it is fast. Having a method to obtain similar measurements on different modalities, such as CT, is of high clinical importance. Numerous studies in the literature depend on the use of the ellipsoid formula and tools that calculate the renal volume based on manually-defined axes are readily available to clinicians (Mayo Clinic, https://www.mayo.edu/research/documents/pkd-center-adpkd-classification/doc-20094754). Obtaining automated, quantitative measurements of those axes, which is the main novelty of our work, is important in order to compare results reported in previous studies.

Our study has limitations. Our training dataset originates from a single institution, and all subjects were scanned using the same protocol. This could possibly drive the models to inadvertently learn and perpetuate biases inherent to the modeled data and hinder the model’s generalizability in unseen cases from different healthcare institutions. Additionally, while we mitigated this by using multiple test sets, the overall size of each of them was not sufficiently large. Although the model was able to perform accurately in images obtained using lower dose levels (Test set 4), an additional study using a larger cohort of dose levels and protocols is required to assess the performance. Furthermore, since our training cohort comprised healthy individuals (potential kidney donors), the models might not be able to extrapolate meaningful clinical descriptors in scenarios involving unhealthy kidneys. A supplementary future study incorporating a general population would shed light on possible failure modes and trends of the models. Moreover, separate automated assessment of renal cortex and medullary volumes is of clinical importance, something that our study did not address. Such a limitation to our work stems from the fact that segmentation of cortex and medulla requires significant labor-intensive and time-consuming manual annotations performed on arterial phase CECT.

In conclusion, the proposed automated segmentation methods can calculate clinical renal descriptors accurately, reliably and promptly using both CE and NC CT images, using only a fraction of the time needed during the manual measurement process. Automated measurements of renal volume and axes, which is something introduced in our work, demonstrate excellent agreement compared to manual measurements and are now a promising candidate to help, verify and guide clinical decision-making.

## Supplementary information

**Additional file 1:** **Supplementary Fig. S1.** Random case from Test set 1. (a1) CECT image and the corresponding manual kidney labels. (a2) CECT image and the corresponding model prediction. (b1) NCCT image and the corresponding manual kidney labels. (b2) NCCT image and the corresponding model prediction. The CE and NC CT images belong to the same CT scan. **Supplementary Fig. S2.** Random case from Test set 1. (a1) CECT image and the corresponding manual kidney labels. (a2) CECT image and the corresponding model prediction. (b1) NCCT image and the corresponding manual kidney labels. (b2) NCCT image and the corresponding model prediction. The CE and NC CT images belong to the same CT scan.

## Data Availability

The datasets used in this study are private and cannot be released publicly. The trained model weights and the code implementation are available from the corresponding author upon reasonable request.

## Abbreviations

3D: Three-dimensional
CE: Contrast-enhanced
CI: Confidence interval
CT: Computed tomography
DSC: Dice similarity coefficient
ICC: Intraclass correlation coefficient
KiTS: Kidney and Kidney Tumor Segmentation Challenge
NC: Noncontrast
PCCT: Photon-counting computed tomography
