# Supplementary material for: Segmentation-based quantitative measurements in renal CT imaging using deep learning
Source: Eur Radiol Exp. 2024 Oct 9;8:110. doi: 10.1186/s41747-024-00507-4 (PMC11465135; doi:10.1186/s41747-024-00507-4)
Supplement: Supplementary file 1 — Additional file 1: Supplementary Fig. S1. Random case from Test set 1. (a1) CECT image and the corresponding manual kidney labels. (a2) CECT image and the corresponding model prediction. (b1) NCCT image and the corresponding manual kidney labels. (b2) NCCT image and the corresponding model prediction. The CE and NC CT images belong to the same CT scan. Supplementary Fig. S2. Random case from Test set 1. (a1) CECT image and the corresponding manual kidney labels. (a2) CECT image and the corresponding model prediction. (b1) NCCT image and the corresponding manual kidney labels. (b2) NCCT image and the corresponding model prediction. The CE and NC CT images belong to the same CT scan. [file 41747_2024_507_MOESM1_ESM.pdf]

Segmentation-based quantitative measurements in renal CT imaging using deep learning

ELECTRONIC SUPPLEMENTARY MATERIAL

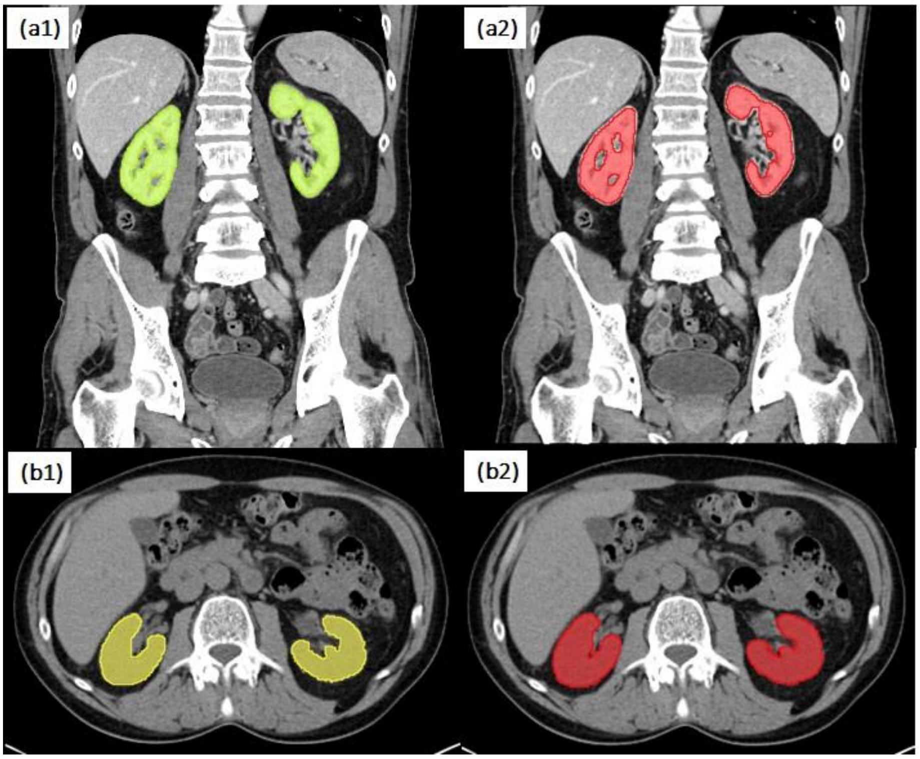

FigureS 1. Random case from Test set 1. (a1) CECT image and the corresponding manual kidney labels. (a2) CECT image and the corresponding model prediction. (b1) NCCT image and the corresponding manual kidney labels. (b2) NCCT image and the corresponding model prediction. The CE and NC CT images belong to the same CT scan.

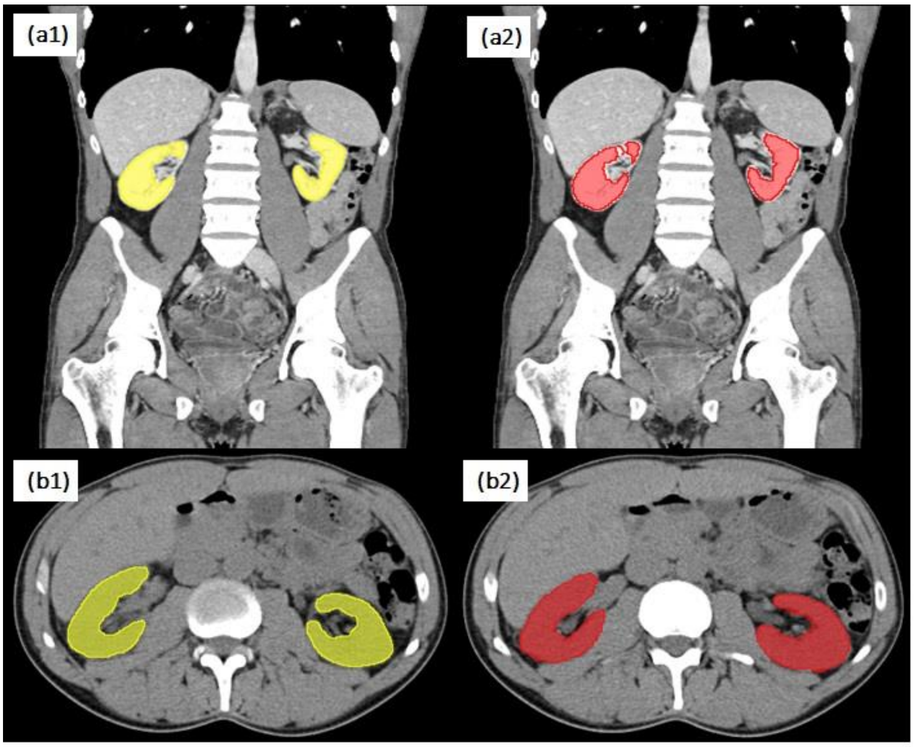

Figure S 2. Random case from Test set 1. (a1) CECT image and the corresponding manual kidney labels. (a2) CECT image and the corresponding model prediction. (b1) NCCT image and the corresponding manual kidney labels. (b2) NCCT image and the corresponding model prediction. The CE and NC CT images belong to the same CT scan

Dataset 1 protocol

The CT scanning protocol used for potential kidney donors included a quality reference mAs of 147 with tube current modulation enabled, a rotation time of the CT gantry of 0.5 s, a pitch of 0.6, collimation of 196x0.6 mm, and a soft tissue reconstruction kernel (Br40, Siemens Healthineers, Germany). Noncontrast images were acquired first. For contrast enhancement, 100mL of iodinated contrast agent (Visipaque 320 mg iodine/mL, GE Healthcare, United States) was administered at a rate of 3 mL/s. Venous phase images were acquired 90 seconds after the start of the injection.

Test set 3 [PCCT] protocol

The scanning protocol used to obtain the PCCT dataset included the following parameters: tube voltage of 120 kVp, CARE kV IQ level 145 (proportional to quality reference mAs), a collimation of 144x0.4 mm, a pitch of 0.8, and a rotation time of the CT gantry of 0.5 s. A soft tissue reconstruction kernel was applied. For contrast enhancement, between 70-100 mL of an iodinated contrast agent (Visipaque 320 mg iodine/mL, GE Healthcare, United States) was administered at a rate of 3 mL per second, with venous phase images acquired 90 seconds after the start of the injection. Virtual Monoenergetic Images (VMIs) were reconstructed at 60 and 190 keV using a software provided by the vendor (syngo.Via).
